# Medical Nutrition Therapy with Meal Sequence and Divided Carbohydrate Intake: Associations with Ketonuria Resolution and Neonatal Outcomes in Gestational Diabetes Mellitus

**DOI:** 10.3390/nu18162665

**Published:** 2026-08-14

**Authors:** Saeko Imai, Shizuo Kajiyama, Yuki Higuchi, Shintaro Kajiyama, Yoshitaka Hashimoto, Michiaki Fukui

**Affiliations:** 1 Department of Food and Nutrition, Kyoto Women’s University, 35, Kitahiyoshi-cho, Imakumano, Higashiyama-ku, Kyoto 605-8501, Japan; 2 Kajiyama Clinic, Kyoto Gojyo Clinic Building, 20-1, Higashionmaeda-cho, Nishinanajyo, Shimogyo-ku, Kyoto 600-8898, Japan; kajiyama-clinic@dream.ocn.ne.jp (S.K.); kaji20091025abcd@gmail.com (S.K.); 3 Department of Endocrinology and Metabolism, Graduate School of Medical Science, Kyoto Prefectural University of Medicine, 465 Kajii-cho, Kawaramachi-Hirokoji, Kamigyo-ku, Kyoto 602-8566, Japan; 4 Osaka Metropolitan University Hospital, 1-5-7, Asahi-cho, Abeno-ku, Osaka 545-0051, Japan; yh14372002@gmail.com; 5 Department of Diabetes and Endocrinology, Matsushita Memorial Hospital, 5-55, Sotojima-cho, Morigu-chi-shi, Osaka 570-8540, Japan; y-hashi@koto.kpu-m.ac.jp; 6 Osaka General Hospital of West Japan Railway Company, 1-2-22, Matsuzaki-cho, Abeno-ku, Osaka 545-0053, Japan; michiaki@koto.kpu-m.ac.jp

**Keywords:** gestational diabetes mellitus, diabetes mellitus, medical nutrition therapy, ketonuria, dietitian, meal-sequence, food order, divided meal, neonatal, vegetable

## Abstract

**Background/Objectives:** Ketonuria is frequently observed in women with gestational diabetes mellitus (GDM), particularly when energy and carbohydrate intake is inadequate. This retrospective study evaluated nutritional status and neonatal outcomes following dietitian-led medical nutrition therapy (MNT) in 517 Japanese women with GDM with and without ketonuria. **Methods:** Dietary counseling emphasized a meal sequence in which vegetables were consumed before protein-rich foods and carbohydrates were consumed last, together with divided carbohydrate intake (4–5 meals/day), to attenuate postprandial glucose excursions while ensuring adequate energy and carbohydrate intake. Clinical characteristics, dietary intake, and neonatal outcomes were compared between women with and without ketonuria. **Results:** Among the participants, 143 (27.7%) exhibited ketonuria. The 60-min plasma glucose concentration during the OGTT was significantly higher in the ketonuria-positive group, whereas age, pre-pregnancy BMI, HbA1c, and glycated albumin did not differ between groups. Energy and carbohydrate intakes were significantly lower in the ketonuria-positive group before and after GDM diagnosis. Following GDM diagnosis, dietary intake decreased further in both groups; however, after MNT, energy and carbohydrate intakes increased significantly, and ketonuria resolved in all affected women. Birth weight, cesarean section rates, and the prevalence of congenital anomalies did not differ significantly between groups. None of the participants subsequently required insulin therapy during pregnancy. **Conclusions:** These findings suggest that dietitian-led MNT incorporating meal sequence and divided carbohydrate intake was associated with improved nutritional adequacy and resolution of ketonuria, with favorable neonatal outcomes observed in Japanese women with GDM regardless of urinary ketone status.

## 1. Introduction

Ethnic and racial differences have been recognized as strong risk factors for gestational diabetes mellitus (GDM). The prevalence of GDM varies considerably across regions, with the highest prevalence reported in the Middle East and North Africa, followed by the Western Pacific region, which includes Japan [1]. These ethnic and regional differences in GDM prevalence may be attributable to variations in body composition, lifestyle factors such as dietary habits and physical activity, and healthcare systems. Compared with Western populations, East Asians, including Japanese, generally have lower BMI values but are more prone to visceral fat accumulation, which may contribute to insulin resistance and pancreatic β-cell dysfunction [2,3,4,5]. These characteristics may partly explain the increasing prevalence of GDM among Japanese women [6], approximately one in eight pregnant women is diagnosed with GDM [7], and this increase has been linked to demographic changes, including delayed marriage and a progressive rise in maternal age at pregnancy and childbirth [8,9]. Particularly, approximately 20% of Japanese women in their twenties are underweight (BMI < 18.5 kg/m^2^), representing one of the highest rates among developed countries [10]. Pregnant women who are underweight are often in a state of undernutrition, which may impair fetal growth and increase the risk of delivering low-birth-weight infants [10,11]. The prevalence of low-birth-weight infants in Japan is approximately 9.4%, which remains high compared with other countries [12].

Based on the concept of the Developmental Origins of Health and Disease (DOHaD), fetal undernutrition has been suggested to increase the future risk of lifestyle-related diseases, such as type 2 diabetes mellitus (T2DM) and obesity in offspring [13]. Low-birth-weight infants are exposed to undernutrition in utero and may subsequently experience postnatal overnutrition after birth, thereby increasing the risk of developing obesity, coronary artery disease, T2DM, and other metabolic disorders later in life [14,15]. Therefore, it is essential to suppress maternal hyperglycemia through dietary therapy while ensuring adequate nutritional intake necessary for optimal fetal growth and development in women with GDM [16,17,18]. However, some women with GDM excessively restrict carbohydrate intake because of fear of postprandial hyperglycemia, resulting in the positive urinary ketone bodies.

Ketone bodies are considered teratogenic substances, and it has been reported that hyperketonemia may promote fetal malformations [19]. Previous studies have reported that lower maternal carbohydrate intake is associated with an increased risk of low-birth-weight [20]. Dietary composition of pregnant women is related to size of the baby at birth, whereas elevated maternal ketone body concentrations have been associated with adverse fetal and offspring outcomes, including reduced fetal heart rate, lower offspring IQ, and lower mental development scores in children [19,20,21,22].

Based on evidence from our previous studies, we have provided dietary counseling for women with GDM that emphasizes a meal-sequence—consuming vegetables first, followed by protein and then carbohydrates last [23]—as well as divided meal intake in which carbohydrate consumption is distributed across multiple meals. In our previous randomized controlled trial in healthy young women, we demonstrated that dividing carbohydrate intake into five smaller meals significantly reduced glycemic variability and improved glycemic indices compared with the conventional three-meal pattern [24].

To our knowledge, few studies have investigated the effects of medical nutrition therapy (MNT) with meal-sequence and divided carbohydrate consumption on maternal glycemic control, body weight, delivery outcomes, and neonatal outcomes in women with GDM. The present retrospective study aimed to compare GDM women with positive urinary ketone and those with the negative urinary ketone who received our individualized MNT provided by registered dietitians, and to clarify the effects of this unique dietary intervention on maternal and neonatal outcomes.

## 2. Materials and Methods

### 2.1. Subjects

This study included women diagnosed with GDM between 2010 and 2023 at obstetrics and gynecology hospitals in Kyoto, Japan, who subsequently received treatment of MNT by registered dietitians at Kajiyama Clinic, a diabetes specialty clinic in Kyoto, Japan. GDM was diagnosed according to the criteria of the Japan Society of Obstetrics and Gynecology using a 75-g oral glucose tolerance test (OGTT) performed during mid-pregnancy (24–28 weeks of gestation). Women were diagnosed with GDM if one or more of the following plasma glucose thresholds were exceeded: fasting glucose ≥ 92 mg/dL, 1-h glucose ≥ 180 mg/dL, or 2-h glucose ≥ 153 mg/dL [18]. The participants were referred to Kajiyama Clinic, within 1–2 weeks after being diagnosed with GDM at collaborating obstetrics and gynecology hospitals, where individualized MNT was initiated by registered dietitians. As the clinic exclusively manages patients with diabetes, including women with GDM, all participants with GDM underwent standardized follow-up throughout pregnancy. Follow-up visits were generally scheduled at 4-week intervals but were conducted every 2 weeks for women considered to be at higher risk. Women considered to be at high risk, including those with two or three abnormal values on OGTT or/and obesity, were instructed to perform self-monitoring of blood glucose (SMBG) daily and were advised to maintain 2-h postprandial blood glucose levels below 120 mg/dL. This was a retrospective observational study conducted using an opt-out approach, written informed consent was not obtained from individual participants. Instead, study information was disclosed in advance and participants were provided with the opportunity to refuse the use of their data whenever possible. All personal information was anonymized and encrypted, and the obtained data were used solely for research purposes. This study was conducted in accordance with the principles of the Declaration of Helsinki and complied with the Ethical Guidelines for Medical and Health Research Involving Human Subjects issued by the Ministry of Education, Culture, Sports, Science and Technology and the Ministry of Health, Labor and Welfare of Japan. The study protocol was approved by the Clinical Research Ethics Committee of Kyoto Women’s University (Approval No. 2023-52).

### 2.2. Measurements

Anthropometric measurements, including height, body weight, and blood pressure, and laboratory assessments included blood glucose levels, OGTT, HbA1c, glycated albumin (GA), and urinary ketone bodies were obtained from all women with GDM. Body weight was measured using a calibrated digital scale (WB-260A, TANITA Co., Ltd., Tokyo, Japan), and blood pressure was measured after at least 10 min of seated rest using an automated sphygmomanometer (HEM-907, Omron Healthcare Co., Ltd., Kyoto, Japan). At each outpatient visit, blood and urine samples were collected approximately 2 h after breakfast and examined at the clinic. Blood glucose was measured by oxidase method (ADAMS Glucose GA-1171 analyzer, ARKRAY Co., Ltd., Kyoto, Japan) and HbA1c was measured by high-performance liquid chromatography (HPLC) method (ADAMS HA-8180, ARKRAY Co., Ltd., Kyoto, Japan), and GA was measured by an enzymatic method (Nagase Diagnostics Co., Ltd., Tokyo, Japan) on an automated biochemical analyzer at the clinic. Urinary ketone bodies were assessed using reagent test strips according to routine clinical practice using an automated biochemical analyzer at the clinic (AUCTION ELEVEN AE-4020 analyzer, ARKRAY Co., Ltd., Kyoto, Japan).

Participants who showed positive urinary ketone bodies at least once after diagnosis of GDM, as assessed using urine samples collected 2 h after breakfast, were classified into the urinary ketone-positive group (the positive group), whereas those who consistently remained negative after diagnosis of GDM were classified into the urinary ketone-negative group (the negative group).

### 2.3. MNT by Registered Dietitians

All 517 participants attended the clinic 3–6 times during pregnancy and received individualized MNT from registered dietitians at every visit. The target energy intake was determined according to the Japanese clinical guidelines, using 25–30 kcal/kg of standard body weight plus an additional 50–450 kcal/day to account for the increased energy requirements during pregnancy [18]. The same MNT protocol was provided to both the urinary ketone-positive and ketone-negative groups. Dietary counseling emphasized adequate energy intake, a vegetable-first meal sequence [25,26], and divided carbohydrate intake by distributing carbohydrate-containing snacks between breakfast and lunch and between lunch and dinner, while avoiding late-evening snacks, to attenuate postprandial glycemic excursions and ensure adequate nutritional intake [24]. The amount of carbohydrate consumed at each meal ranged from approximately 30 to 70 g, depending on each participant’s estimated energy requirements and dietary prescription.

In addition to counseling on meal sequence and divided carbohydrate intake, individualized nutritional counseling was tailored to each patient’s dietary habits, lifestyle, and work schedule to facilitate adequate intake of energy and other essential nutrients during pregnancy while maintaining appropriate glycemic control. Practical recommendations included simple divided-meal strategies using readily available foods such as rice balls, sandwiches, and soy protein bars, as well as easy-to-prepare vegetable dishes using tomato juice or frozen vegetables and microwave cooking. Patients were also provided with make-ahead meal recipes to facilitate long-term adherence to dietary therapy. Although the same MNT protocol was provided to both the urinary ketone-positive and urinary ketone-negative groups, women with ketonuria often exhibited excessive restriction of carbohydrate intake. Therefore, they received individualized counseling to consume the recommended amount of carbohydrates distributed over 4–5 meals and snacks per day to eliminate ketonuria while maintaining appropriate glycemic control.

Nutrient intake and food group consumption were interviewed and calculated by registered dietitians based on dietary records obtained for pre-pregnancy, after diagnosis, and after MNT at each visit. Participants completed dietary records, which were subsequently reviewed and refined through detailed face-to-face interviews conducted by registered dietitians to improve the accuracy of the recorded food intake. Nutritional analyses were then performed using nutrition analysis software (Smart Nutrition Calculation Ver. 9.2; Ishiyaku Publishers, Tokyo, Japan). The intervention focused exclusively on individualized MNT. Structured exercise therapy was not prescribed, and no oral glucose-lowering agents were used. Women who required insulin therapy at referral were excluded from the present analysis.

### 2.4. Statistical Analysis

Data are presented as the median and interquartile range (IQR) [first quartile (Q1)–third quartile (Q3)] unless otherwise specified. Because the assumptions of normality and homogeneity of variance were not satisfied, nonparametric tests were used for all analyses. Differences between the urinary ketone-positive and the ketone-negative groups were evaluated using the Mann–Whitney U test. Within-group changes were assessed using the Wilcoxon signed-rank test, including comparisons before and after MNT and pairwise comparisons among the three assessment time points: pre-pregnancy, after GDM diagnosis, and following MNT. Statistical significance was defined as a two-sided *p* value < 0.05. All statistical analyses were performed using SPSS software (IBM SPSS Statistics version 28; SPSS Japan Inc., Tokyo, Japan).

## 3. Results

### 3.1. Characteristics of the Participants

**Figure 1:**
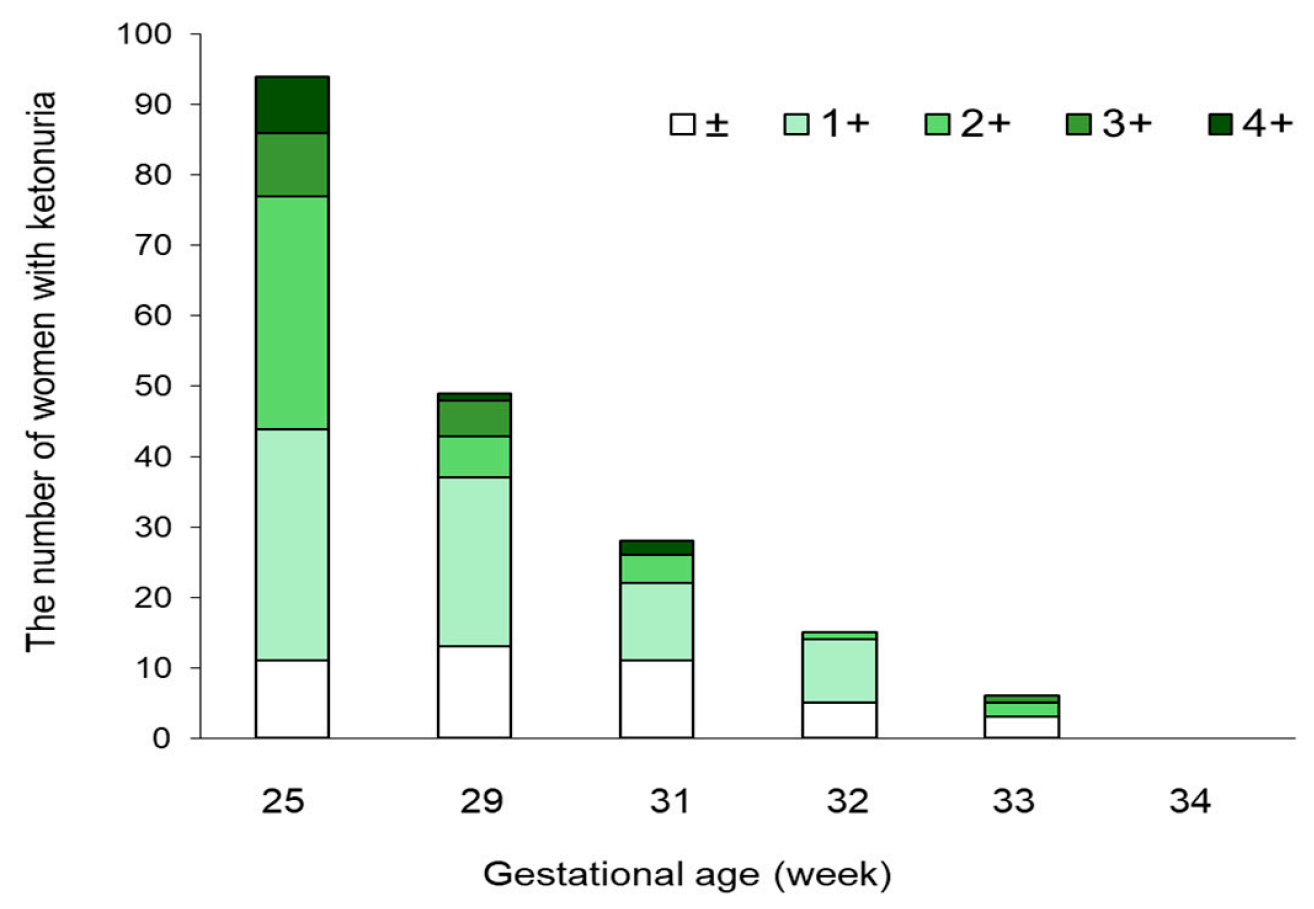
Number of women with GDM who tested positive for urinary ketone bodies according to gestational age during MNT.

Of the 519 women with GDM between 24 and 37 weeks of gestation who received individualized MNT from registered dietitians, two women who required insulin therapy at first visit were transferred to other hospitals were excluded. Consequently, 517 women were included in the final analysis. Of these, 143 women (27.7%) exhibited positive urinary ketone bodies at least once after diagnosis of GDM were classified into positive group, whereas 374 women (72.3%) who consistently tested negative for urinary ketone bodies were classified into the negative group. Baseline characteristics of the women with GDM in the positive and the negative groups are shown in Table 1. No significant differences were observed between the two groups with respect to age, HbA1c, GA, blood pressure, and family history of T2DM, although results of the 75-g OGTT at 60 min were significantly higher in the positive group than in the negative group (*p* = 0.04). Pre-pregnancy BMI did not differ significantly between the positive and the negative groups, however, when participants were categorized according to pre-pregnancy BMI as underweight (BMI < 18.5 kg/m^2^), normal weight (BMI 18.5 to < 25 kg/m^2^), and obesity (BMI ≥ 25 kg/m^2^), the proportion of underweight individuals tended to be higher in the positive group than in the negative group (*p* = 0.098). Figure 1. shows the number of women with GDM who tested positive for urinary ketone bodies according to MNT. Following MNT, the number of women with positive urinary ketone bodies progressively decreased, and ultimately all women became negative.

### 3.2. Changes in Nutrient Intake

**Figure 2:**
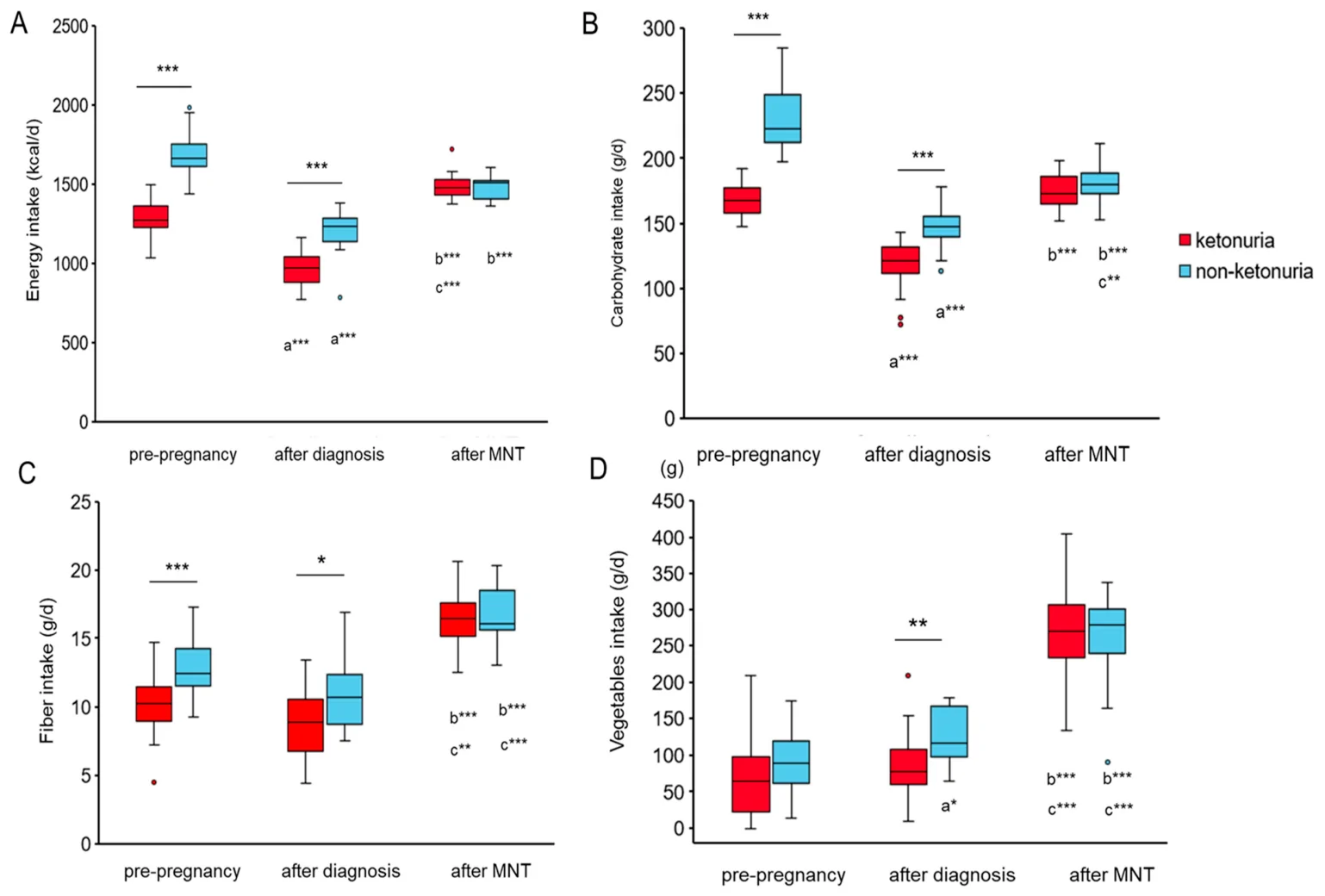
Changes in energy, carbohydrate, dietary fiber, and vegetable intake before pregnancy, after GDM diagnosis, and after medical nutrition therapy (MNT) in women with GDM according to urinary ketone status. (**A**) Energy intake (kcal/day), (**B**) carbohydrate intake (g/day), (**C**) dietary fiber intake (g/day), and (**D**) vegetable intake (g/day). Data are presented as box-and-whisker plots. The horizontal line within each box represents the median, the box represents the interquartile range (IQR), and the whiskers extend to the most extreme values within 1.5 × IQR of the lower and upper quartiles. Individual dots represent outliers beyond 1.5 × IQR. Comparisons between the urinary ketone-positive and urinary ketone-negative groups were performed using the Mann–Whitney U test (* *p* < 0.05, ** *p* < 0.01, *** *p* < 0.001). Within-group comparisons (a, before pregnancy vs. after GDM diagnosis; b, after GDM diagnosis vs. after MNT; and c, before pregnancy vs. after MNT) were performed using the Wilcoxon signed-rank test (* *p* < 0.05, ** *p* < 0.01, *** *p* < 0.001).

Nutrient intake among women with GDM in the positive and the negative groups was compared before pregnancy, after GDM diagnosis, and after MNT (Table 2 and Figure 2A–D). Energy intake significantly decreased from pre-pregnancy to post-diagnosis in both groups, whereas they significantly increased from post-diagnosis to post-MNT (Figure 2A). Between-group comparisons showed that energy intake was significantly lower in the positive group both before pregnancy, and after diagnosis (Figure 2A). Following MNT, energy intake increased in both groups, resulting in no significant difference between the groups. Carbohydrate intake was also lower in the positive group than in the negative group before pregnancy (Figure 2B). After diagnosis, carbohydrate intake declined in both groups, and the positive group continued to consume significantly less carbohydrate than the negative group. After MNT, carbohydrate intake increased significantly in both groups, and the between-group difference was no longer significant. Similarly, dietary fiber intake was significantly lower in the positive group than the negative group before pregnancy and after diagnosis (Figure 2C). After MNT, dietary fiber intake increased significantly in both groups, and no significant difference was observed between the groups. Vegetable intake was low in both groups before pregnancy [positive group: 65 g (28–96); negative group: 90 g (64–118)] and after GDM diagnosis [78 g (60–103) and 118 g (100–156), respectively]. Following MNT, however, vegetable intake increased significantly in both groups to 270 g (239–300) and 271 g (240–299), respectively (Figure 2D). In addition to carbohydrate intake, fat intake decreased significantly following the diagnosis of GDM, although they increased significantly after MNT in both groups (Table 2A,B). Likewise, the intakes of several key micronutrients, including calcium, iron, and vitamins, increased significantly following MNT, indicating an overall improvement in dietary quality.

### 3.3. Maternal and Neonatal Outcomes

After MNT, both HbA1c and GA were maintained within the target range, indicating good glycemic control in both groups without insulin therapy (Table 3). Gestational weight gain was numerically lower in the urinary ketone-positive group than in the urinary ketone-negative group, however, the difference did not reach statistical significance (*p* = 0.093), nevertheless birth weight outcomes did not differ significantly between the two groups. Additionally, no significant differences were observed with respect to mode of delivery and the incidence of congenital abnormalities between two groups.

## 4. Discussion

Unlike Western populations, in which GDM is strongly associated with obesity, Japanese women with GDM often present with relatively low BMI despite a predisposition to visceral fat accumulation and metabolic dysfunction [3,4,5,6]. A recent meta-analysis of Japanese pregnant women reported that women who were underweight before pregnancy (BMI < 18.5 kg/m^2^) had a 1.6-fold higher risk of delivering low-birth-weight infants, a 1.2-fold higher risk of preterm birth, and a 1.6-fold higher risk of small-for-gestational-age infants, with neonatal birth weight being approximately 115 g lower on average [11]. In the present study, the 60-min plasma glucose concentration during OGTT was significantly higher in the ketonuria-positive group. Because the 60-min glucose value is considered to reflect early-phase insulin secretion, this finding may indicate a greater impairment of β-cell function in these women.

Women who experienced greater postprandial glucose excursions may have responded by excessively restricting carbohydrate intake in an attempt to improve glycemic control, which may have contributed to the development of ketonuria. Furthermore, ketone bodies may increase during late pregnancy because of enhanced lipolysis and increased fetal energy demands, and may also occur as a consequence of maternal energy and carbohydrate restriction [27,28]. Previous studies have suggested that maternal hyperketonemia may adversely affect offspring development, and elevated maternal β-hydroxybutyrate and free fatty acid levels during late pregnancy were inversely associated with IQ scores in children aged 2–5 years [22]. However, evidence regarding the long-term effects of maternal ketonemia on offspring remains limited, and the underlying mechanisms have not yet been fully elucidated.

In the present study, repeated MNT provided throughout the second and third trimesters appeared to improve maternal nutrient intake. Furthermore, no participants remained positive for urinary ketones after MNT, and insulin therapy was not required in any case during pregnancy. Notably, the prevalence of low-birth-weight infants (<2500 g) did not differ significantly between the urinary ketone-positive and ketone-negative groups and was lower than the national prevalence of 9.4% among all live births in Japan in 2019 [12]. No significant differences were observed between the ketone-positive and ketone-negative groups in the prevalence of low-birth-weight infants/macrosomia, cesarean section rates, and the prevalence of congenital abnormalities and they were lower than the average rates reported among pregnant women in Japan. These findings suggest that continuous nutritional intervention may have contributed to maintaining appropriate fetal growth, even among patients who had experienced urinary ketone positivity during pregnancy.

Maintaining adequate energy and carbohydrate intake without excess or deficiency is particularly important in women with GDM to support both maternal metabolic health and fetal growth [16,17,18]. However, achieving this balance is often challenging in clinical practice. In addition to concerns about postprandial hyperglycemia and the possibility of insulin therapy [29], many women experience considerable physical and psychological burdens during pregnancy, including nausea and vomiting [30], anxiety regarding fetal growth and pregnancy outcomes, and difficulties adapting to dietary modifications required for glycemic management. These factors may inadvertently lead to inappropriate dietary restriction and inadequate nutrient intake. Most women are employed and lead busy lives, they may have difficulty devoting substantial time and effort to dietary therapy. Consequently, complicated and time-consuming dietary approaches, such as weighing foods and calculating energy and carbohydrate intake, are often difficult to maintain in daily practice. Under these circumstances, our MNT strategy incorporating meal-sequence and divided carbohydrate intake may provide a simple and practical approach for the nutritional management of GDM. In addition to attenuating postprandial glucose excursions, this strategy may facilitate near-recommended intakes of energy, carbohydrates, and other essential nutrients during pregnancy, which may contribute to both optimal maternal metabolic control and healthy fetal growth. The 2025 American Diabetes Association (ADA) guidelines and previous reports recommend a minimum carbohydrate intake of 175 g/day during pregnancy to ensure appropriate fetal growth and brain development [16]. Markedly, carbohydrate intake in both groups increased following MNT and nearly reached this recommended threshold, indicating that our nutritional intervention may have contributed to improving dietary adequacy while maintaining glycemic control.

As reported in our previous studies, meal-sequence may attenuate postprandial glucose excursions by several physiological mechanisms [23,26]. One of the mechanism is dietary fiber contained in vegetables consumed first delays the digestion and absorption of carbohydrates consumed later in the meal [31]. Subsequent intake of protein and fat stimulates the secretion of incretin hormones such as glucagon-like peptide-1 (GLP-1) and glucose-dependent insulinotropic polypeptide (GIP), prolongs gastric emptying time, delays nutrient delivery to the small intestine, and promotes timely insulin secretion [32]. Moreover, implementation of the meal-sequence strategy markedly increased vegetable intake in all women following MNT in this study.

Previous studies have suggested that women with GDM who consume snacks or distribute their food intake across multiple eating occasions are more likely to achieve glycemic targets than those with less frequent eating patterns [33]. We previously demonstrated that dividing dinner carbohydrate intake into two separate eating occasions attenuated glycemic variability and reduced postprandial insulin and free fatty acid responses in both healthy young adults [34] and patients with type 2 diabetes [35,36]. These findings support the potential benefits of carbohydrate distribution as a strategy for improving metabolic control [33,34,35,36]. Consistent with these findings, the divided-carbohydrate approach used in the present study may have helped attenuate postprandial glucose excursions while enabling participants to achieve adequate energy and nutrient intake.

To achieve adequate nutritional intake during pregnancy, MNT should incorporate not only meal-sequence and divided meal strategies but also individualized guidance tailored to each patient’s clinical condition, dietary habits, and lifestyle. Providing practical food-based recommendations may facilitate adherence and help ensure appropriate energy and nutrient intake. For example, we recommended a simple and feasible divided-meal strategy, such as consuming tomato juice prior to the rice balls or sandwiches as snacks between breakfast and lunch and between lunch and dinner [34,35,36]. We also provided recipes for simple, palatable vegetable dishes that could be easily prepared using a microwave. These approach enabled women to achieve the recommended daily energy, carbohydrate, and other nutritional intake while minimizing postprandial hyperglycemia, which may have contributed to favorable maternal and neonatal outcomes in this study.

Several limitations of this study should be acknowledged. First, long-term outcomes of both the offspring and mothers were not assessed. Although neonatal birth weight and congenital abnormalities were evaluated, subsequent growth and neurodevelopment of the children were not examined. In addition, maternal metabolic outcomes after delivery, including the development of T2DM, were not investigated [37]. Therefore, future longitudinal studies are warranted to determine the long-term impact of maternal urinary ketone positivity on offspring health and development, as well as on maternal metabolic health after pregnancy. Second, because this was a retrospective observational study, residual confounding cannot be excluded. Although the urinary ketone-positive and ketone-negative groups were compared with respect to clinical characteristics, dietary intake, and neonatal outcomes, multivariable analyses were not performed to adjust for potential confounding factors. Therefore, the observed improvements in nutritional intake and the favorable neonatal outcomes cannot be attributed solely to the MNT intervention. Prospective studies with appropriate multivariable adjustment and, ideally, randomized controlled designs are warranted to confirm the effects of this nutritional approach. Third, the present study compared maternal and neonatal outcomes between ketone-positive and ketone-negative women with GDM who received our MNT based on meal-sequence and divided meal intake. However, to more clearly evaluate the effectiveness of this dietary approach, future studies should compare our intervention with conventional MNT methods, such as using the Food Exchange Lists for Diabetes [38] or low glycemic index (GI) [39]. Fourth, blood test including blood glucose, HbA1c, and GA levels were measured at each clinical visit, detailed glycemic variability assessed by continuous glucose monitoring system (CGM) could not be evaluated. Future studies using CGM are currently being planned to enable more objective and comprehensive assessment of postprandial glucose fluctuations. Fifth, dietary intake assessment in women with GDM relied on self-reported dietary records and interviews conducted by registered dietitians. Although dietary intake pre-pregnancy, after diagnosis, and after MNT was estimated based on these dietary records and interviews by registered dietitians, participants were not strictly instructed to weigh all foods because such procedures would impose a substantial burden during pregnancy. Discrepancies between reported and actual intake may have occurred. Future studies should incorporate more objective dietary assessment methods, such as food frequency questionnaires (Food Frequency Questionnaire, FFQs) and artificial intelligence (AI) –based applications capable of estimating nutrient intake from meal photographs. Additionally, as this was a single-center study, future large-scale multicenter investigations are required to establish the external validity of our findings and to further evaluate the effectiveness of this unique MNT approach.

## 5. Conclusions

In conclusion, following dietitian-led MNT incorporating a vegetable-first meal sequence and divided carbohydrate intake, nutritional adequacy improved and ketonuria was no longer observed in women who had previously exhibited ketonuria. Favorable neonatal outcomes were observed in both urinary ketone-positive and ketone-negative groups, and none of the participants subsequently required insulin therapy during pregnancy. These findings support the potential clinical utility of this practical nutritional approach; however, prospective studies with appropriate adjustment for potential confounders are needed to establish its effectiveness.

Preliminary findings from this study were presented in abstract form at the EASD 2025 (European Association for the Study of Diabetes, in Sep. *Diabetologia* **2025**, *68* (Suppl 1): S1–S754, SO68 781. https://link.springer.com/article/10.1007/s00125-025-06497-1#Sec116 (accessed on 5 May 2026)).

## Figures and Tables

**Table 1:** Characteristic in women with GDM with and without urinary ketone (*n* = 517).

	All (*n* = 517)	Ketone-Positive Group (*n* = 143)	Ketone-Negative Group (*n* = 374)	*p*
Height (cm)	158 (155–162)	158 (155–162)	158 (155–162)	0.798
Age (years)	33.0 (30.0–36.0)	33.0 (30.0–36.0)	33.0 (29.0–36.0)	0.596
Pre-pregnancy BW (kg)	52.0 (47.0–58.0)	52.0 (47.0–57.0)	52.0 (47.0–58.0)	0.306
Pre-pregnancy BMI (kg/m^2^)	20.7 (19.1–22.8)	20.3 (18.7–22.7)	20.7 (19.3–22.9)	0.165
<18.5 *n* (%)	85 (16.4)	31 (21.7)	54 (14.4)	0.098
<18.5 < 25.0 *n* (%)	362 (70.0)	91 (63.6)	271 (72.7)	
<25.0 *n* (%)	70 (13.5)	21 (14.7)	49 (12.8)	
75-g OGTT 0 min (mg/dL)	90 (81–96)	88 (81–95)	90 (81–97)	0.184
60 min (mg/dL)	177 (156–191)	181 (160–193)	176 (154–191)	0.040
120 min (mg/dL)	153 (131–166)	154 (132–169)	153 (130–165)	0.304
HbA1c (%)	5.20 (5.10–5.40)	5.20 (5.10–5.40)	5.20 (5.00–5.40)	0.956
GA (%)	13.3 (12.5–14.1)	13.3 (12.7–14.0)	13.4 (12.4–14.3)	0.644
SBP (mmHg)	103 (97–110)	103 (97–112)	103 (96–110)	0.518
DBP (mmHg)	60 (55–64)	61 (97–112)	60 (55–65)	0.126
Family history of T2DM *n* (%)	216 (41.8)	55 (38.5)	161 (43.0)	0.344

Data are presented as the median and interquartile range IQR (Q1–Q3) or *n* (%). BW; body weight, BMI; body mass index, OGTT; oral glucose tolerance test, GA; glycated albumin, SBP; systolic blood pressure, DBP; diastolic blood pressure, T2DM; type 2 diabetes mellitus.

**Table 2:** (**A**) Changes in nutritional intake before pregnancy, after GDM diagnosis, and after MNT in women with GDM who exhibited urinary ketone positivity. (**B**) Changes in nutritional intake before pregnancy, after GDM diagnosis, and after MNT in women with GDM who exhibited urinary ketone negativity.

(A)			
	Nutritional Intake		
	Pre-Pregnancy	After Diagnosis	After MNT
Energy (kcal)	1275 (1232–1356)	978 (892–1032) a***	1481 (1448–1533) b***c***
protein (g)	45.0 (41.9–52.5)	43.4 (38.1–46.3)	68.0 (64.4–75.2) b***c***
Fat (g)	51.9 (46.5–59.2)	37.5 (31.7–46.0) a***	64.0 (59.9–68.7) b***c**
Carbohydrate (g)	168.0 (159.7–177.2)	122.0 (99.8–123.6) a***	173.0 (165.7–186.5) b***
Dietary fiber (g)	10.3 (9.0–11.3)	8.9 (6.8–10.4)	16.5 (15.6–17.5) b***c**
Salt (g)	6.0 (4.8–6.7)	5.5 (4.3–6.2) a*	8.3 (7.5–9.6) b***c**
Calcium (mg)	278 (148–328)	163 (94–283) a**	439 (350–544) b***c***
Iron (mg)	4.0 (3.3–4.6)	3.4 (2.9–4.2)	6.8 (6.3–7.8) b**c*
Vitamin B1 (mg)	0.49 (0.42–0.63)	0.43 (0.38–0.54)	1.00 (0.83–1.12) b***c***
Vitamin B12 (µg)	2.4 (1.7–5.8)	2.1 (1.5–5.1)	5.5 (3.0–10.4) b**c**
Folic acid (µg)	117 (96–159)	108 (76–172)	273 (239–318) b***c***
Vitamin C (mg)	26 (13–36)	26 (16–34)	80 (59–93) b***c***
**(B)**			
	**Nutritional Intake**		
	**Pre-Pregnancy**	**After Diagnosis**	**After MNT**
Energy (kcal)	1667 (1619–1737)	1235 (1143–1280) a***	1512 (1424–1528) b***
protein (g)	61.2 (54.2–67.8)	53.1 (49.4–54.9) a**	66.2 (60.1–70.9) b***
Fat (g)	63.4 (57.5–71.4)	49.6 (46.2–59.1) a**	64.5 (60.0–68.8) b***
Carbohydrate (g)	223.0 (213.3–236.0)	149.9 (142.5–156.4) a***	180.7 (173.1–187.8) b***c**
Dietary fiber (g)	12.5 (11.7–13.9)	10.7 (9.1–12.0)	16.1 (15.7–18.1) b***c***
Salt (g)	8.1 (6.9–9.2)	7.0 (5.1–8.0)	9.1 (7.2–10.1) b**
Calcium (mg)	383 (302–556)	343 (215–411)	435 (372–568) b***c**
Iron (mg)	5.0 (4.1–5.8)	4.4 (3.6–5.3)	7.2 (6.2–7.7) b***c**
Vitamin B1 (mg)	0.69 (0.59–0.97)	0.61 (0.50–0.76)	0.97 (0.82–1.08) b***c**
Vitamin B12 (µg)	2.4 (1.8–4.9)	2.8 (2.2–5.3)	4.9 (2.2–7.3) b**c***
Folic acid (µg)	184 (126–220)	173 (136–190)	283 (244–316) b***c***
Vitamin C (mg)	29 (27–50)	44 (37–52)	90 (60–102) b***c***

(**A**) Data are presented as the median and interquartile range IQR (Q1–Q3). Within-group comparisons (a, before pregnancy vs. after GDM diagnosis; b, after GDM diagnosis vs. after MNT; and c, before pregnancy vs. after MNT) were performed using the Wilcoxon signed-rank test (* *p* < 0.05, ** *p* < 0.01, *** *p* < 0.001). (**B**) Data are presented as the median and interquartile range IQR (Q1–Q3). Within-group comparisons (a, before pregnancy vs. after GDM diagnosis; b, after GDM diagnosis vs. after MNT; and c, before pregnancy vs. after MNT) were performed using the Wilcoxon signed-rank test (** *p* < 0.01, *** *p* < 0.001).

**Table 3:** Maternal and neonatal outcome after MNT in women with GDM with and without urinary ketonuria (*n* = 517).

	All (*n* = 517)	Ketone-Positive Group (*n* = 143)	Ketone-Negative Group (*n* = 374)	*p*
HbA1c (%)	5.40 (5.20–5.60)	5.40 (5.20–5.60)	5.40 (5.20–5.60)	0.467
GA (%)	13.0 (12.3–13.7)	12.8 (12.1–13.7)	13.2 (12.4–13.7)	0.207
BW at delivery (kg)	59.6 (54.6–66.6)	58.8 (54.0–65.8)	59.8 (55.3–67.2)	0.261
Weight gain during pregnancy (kg)	7.2 (5.1–9.3)	6.8 (4.3–9.0)	7.3 (5.3–9.6)	0.093
Birth weight (g)	3032 (2752–3285)	3072 (2818–3275)	3013 (2740–3289)	0.327
Low-birth-weight infant *n* (%)	38 (7)	12 (8)	26 (7)	0.589
Normal-weight infant *n* (%)	473 (92)	128 (90)	345 (92)	
Macrosomia *n* (%)	6 (1)	3 (2)	3 (1)	
Caesarean *n* (%)	70 (13.5)	18 (12.6)	52 (13.9)	0.696
Congenital anomalies *n* (%)	16 (3.1)	3 (2.1)	13 (3.5)	0.256

Data are presented as the median and interquartile range IQR (Q1–Q3) or *n* (%). BW; body weight, GA; glycated albumin.

## Data Availability

The data are not publicly available due to the privacy reasons. Data supporting the reported results are available upon reasonable request and in accordance with the ethical principles.
